# Phase II study of rituximab given in conjunction with standard chemotherapy in primary central nervous system lymphoma (PCNSL): a trial of the ECOG-ACRIN cancer research group (E1F05)

**DOI:** 10.18632/oncotarget.22332

**Published:** 2017-11-06

**Authors:** Lode J. Swinnen, Anne O’Neill, Philip H. Imus, Sachin Gujar, David Schiff, Lawrence R. Kleinberg, Ranjana H. Advani, Erin M. Dunbar, Dennis Moore, Stuart A. Grossman

**Affiliations:** ^1^ Johns Hopkins University, Baltimore, Maryland, USA; ^2^ Dana Farber Cancer Institute, Boston, Massachusetts, USA; ^3^ University of Virginia, Charlottesville, Virginia, USA; ^4^ Stanford Cancer Institute, Stanford, California, USA; ^5^ University of Florida, Gainesville, Florida, USA; ^6^ Wichita NCORP, Wichita, Kansas, USA

**Keywords:** primary CNS lymphoma, rituximab, high-dose methotrexate, blood-brain barrier, PCNSL

## Abstract

**Background:**

Therapy of primary CNS lymphoma (PCNSL) has focused on multi-agent chemotherapy designed to cross the blood brain barrier. Rituximab has demonstrated activity in PCNSL. E1F05 is an ECOG-ACRIN multicenter phase 2 prospective trial of rituximab with high-dose methotrexate (HD-MTX)-based chemotherapy similar to the RTOG 93-10 regimen, omitting radiotherapy.

**Methods:**

Immunocompetent patients with newly diagnosed PCNSL received HD-MTX 3.5g/m2 with vincristine every two weeks for 5 doses; procarbazine for 7 days in weeks 1, 5, and 9; cytarabine 3g/m2/day IV for 2 days in weeks 11 and 14; a dexamethasone taper over 6 weeks; and rituximab 375mg/m2 IV infusion 3 times per week for weeks 1-4. Subjects with CSF involvement received intrathecal methotrexate 12mg every two weeks.

**Results:**

Twenty-six patients were enrolled; median age was 57. Sixteen subjects (65%) completed treatment per protocol; the most common reason for discontinuation was adverse events, and 2 subjects discontinued due to progressive disease (PD). Complete response (CR) + unconfirmed CR (CRu) was 16/25 (64%), overall response rate was 20/25 (80%), and 4/25(16%) had PD as best response. Median progression free survival (PFS) was 34 months, and median overall survival has not been reached at 40 months’ median follow up. Two year PFS was 63%. The most common grade 3-4 toxicities were hematologic.

**Conclusion:**

The addition of rituximab to multi-agent chemotherapy is well tolerated. Outcomes are comparable to or better than those seen in RTOG 93-10, which included RT. These and other results suggest rituximab has activity in the CNS. [ECOG-ACRIN E1F05]

**Clinical Trial Registration:**

NCT00335140, clinicaltrials.gov

## INTRODUCTION

Primary central nervous system lymphoma (PCNSL) is an aggressive lymphoma with a median survival of 1 to 3 months if not treated. Surgical resection has only minimal effects on survival [1]. Radiation therapy alone results in a high response rate, but a short median survival of 12 months due to disease relapse [2]. The addition of the standard systemic lymphoma chemotherapy (CHOP) to radiation has not improved median survival [3]. Chemotherapy regimens designed to penetrate the blood brain barrier (BBB), mainly high-dose methotrexate (HD-MTX), with subsequent radiation therapy, resulted in a median overall survival of 42 months, and five year survival of 26% [4, 5]. These initial findings were confirmed by the RTOG/SWOG-9310 study of combined modality therapy [6].

Although median survival has been improved with such combined modality therapy, relapse remains common and late neurologic toxicity has emerged as a major problem. Delayed leukoencephalopathy - a syndrome of dementia, gait disturbance, and incontinence, with concurrent cerebral volume loss and deep white matter changes on MRI - is believed to result from exposure to both cranial irradiation and methotrexate [7-9]. Long term follow up on the original cohort of patients treated with the combined modality regimen subsequently studied in RTOG/SWOG-9310 showed that nearly a third of patients under the age of 60 and essentially all patients over the age of 60 experienced severe late treatment-related neurotoxicity [10].

A strategy that would both intensify anti-lymphoma therapy and avoid the use of radiation would therefore be very attractive. The addition of rituximab to chemotherapy regimens for virtually all systemic B-cell lymphoma has resulted in improved efficacy with minimal side effects. The durable remission rate for systemic aggressive lymphoma increased from 35% to 53% in a large randomized trial with the addition of rituximab, the only real improvement in the initial therapy of aggressive lymphoma since the development of CHOP chemotherapy [11]. Although rituximab is not expected to cross the intact BBB, many observations support a response in parenchymal brain lesions when the monoclonal antibody is used as monotherapy. In patients with recurrent PCNSL rituximab monotherapy produced sustained remissions [12]. Several studies have since suggested a benefit of rituximab in both relapsed and newly diagnosed PCNSL [12-16]. Rituximab has been detected in the CSF of human subjects in other diseases, typically at concentrations between 2 and 3 logs below that of the serum [17, 18].

We performed an ECOG-ACRIN multi-center phase II single-arm trial of rituximab added to the chemotherapy backbone used in RTOG/SWOG-9310, without radiation, for initial therapy of PCNSL. The rituximab was given at high doses during initial treatment. We hypothesized that this is when BBB breakdown in the tumor microenvironment is greatest, and higher serum levels at that time would lead to higher parenchymal levels and therefore clinical activity, based partly on pharmacokinetic studies performed in trials of high-dose rituximab in systemic B cell malignancies [19].

## RESULTS

Twenty-six patients across 11 institutions were enrolled from December 2006 to February 2010 and accrual was suspended for a pre-defined evaluation of outcome. Although this study met its first stage response objective to continue accrual into a second stage, given the potential for continued slow accrual in a second stage, the study was closed and the final study results are reported here.

One subject was deemed ineligible because NHL histology could not be confirmed; this subject’s data are excluded from baseline and outcome summaries, but included in the evaluation of toxicity since they began protocol treatment.

### Patient and disease characteristics

Subject characteristics are presented in Table 1. The median age was 57 (range, 30 to 76), and 40% of subjects were male. Thirty-six percent of subjects had an ECOG PS of 2 or 3.

**Table 1:** Subject baseline characteristics

Characteristics		N	(%)
Gender	Male	10	(40)
	Female	15	(60)
Race	White	24	(96)
	Other	1	(4)
Age	Minimum	30	-
	25%	51	-
	Median	57	-
	75%	68	-
	Maximum	76	-
Age <60		15	(60)
Age ≥ 60		10	(40)
ECOG PS	0-1	16	(64)
	2-3	9	(36)
Neurologic Function Status	No Symptoms	-	-
	Minor Symptoms	11	(44)
	Moderate Symptoms/Fully Active	5	(20)
	Moderate Symptoms/Less than Fully Active	7	(28)
	Severe Neurologic Symptoms	2	(8)

### Treatment compliance

Sixteen subjects among 25 eligible (65%) completed treatment per protocol. The reasons for discontinuation for the other 9 were: adverse events, side effects, or complications of therapy (4 subjects); progressive disease (2 subjects); patient withdrawal/refusal (2 subjects), other (1 subject). Median time on protocol treatment was 14 weeks (range 4-19).

### Toxicity

No treatment-related deaths occurred. The most common grade 3-4 toxicities were hematologic (Table 2). Five subjects of 26 who began protocol treatment experienced at worst a grade 3 toxicity (19%), and 19 subjects experienced at worst a grade 4 toxicity (73%). Four subjects stopped treatment early due to adverse events: one for a bowel perforation (during week 6 of treatment, while still on dexamethasone); one for acute renal failure; and two for other reasons.

**Table 2:** Grade 3 and 4 toxicities that occurred in more than 10% of subjects

Toxicity	Grade 3	Grade 4
Anemia	5	1
Lymphopenia	13	4
Neutropenia	4	16 any febrile neutropenia
Thrombocytopenia	3	2
Fatigue	4	-
ALT elevation	5	2
AST elevation	3	1
Hypokalemia	8	-
Motor Neuropathy	3	-
Sensory Neuropathy	2	1
Thrombosis/Embolism venous?	3	-

### Disease response

The rate of CR + CRu was 16/25 (64%, 95% CI 42.5-82%). The overall response rate was 80% (20/25) (95% CI: 59.2-93.2%); 16% (4/25) had progressive disease while on therapy (Table 3).

**Table 3:** Response rates

Response	n (%)
CR	7 (28)
CRU^*^	9 (36)
PR	4 (16)
Stable	--
PD	4 (16)
Unevaluable	1 (4)
Total	25

CR, complete response; CRu, Complete response, unconfirmed; PR, partial response; PD, progressive disease.

### Secondary endpoints

**Figure 1:**
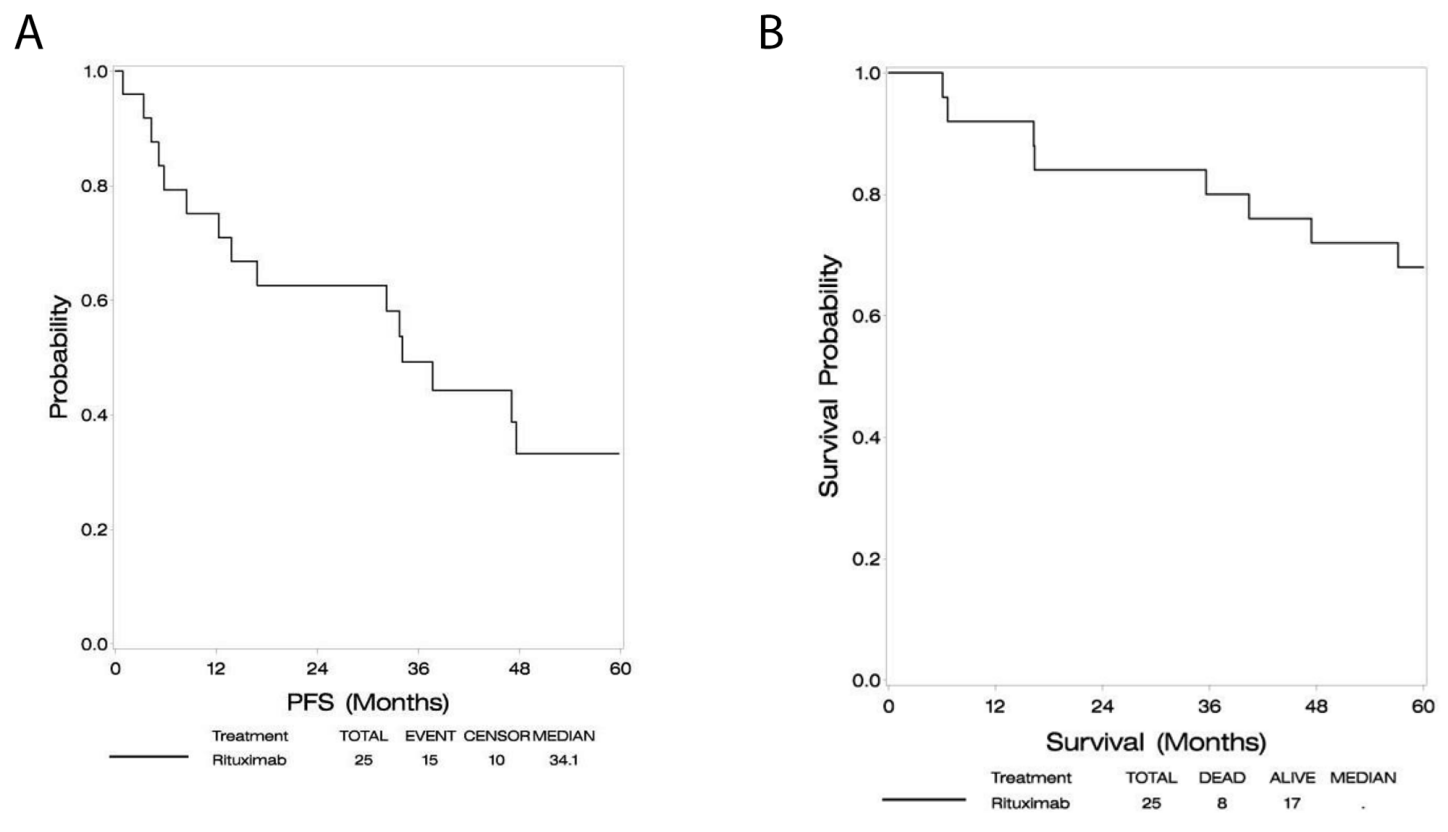
Progression free survival **(A)**, overall survival **(B)**.

At a median follow-up of 60 months, the median PFS was 34.1 months (95% CI: 12.2-60.1+), and median OS had not been reached as depicted in Figures 1A and 1B. Two year PFS was 63%. In an exploratory analysis, subjects older than 60 had similar progression free survival but a trend toward inferior overall survival compared with subjects younger than age 60.

### Rituximab concentrations

Nine (36%) subjects had positive CSF cytology, but 12 received intrathecal therapy (the other three received treatment given a high clinical suspicion of CSF involvement). Of the twelve subjects who received intrathecal, 7 had at least one sample sent for rituximab concentrations in CSF.

**Figure 2:**
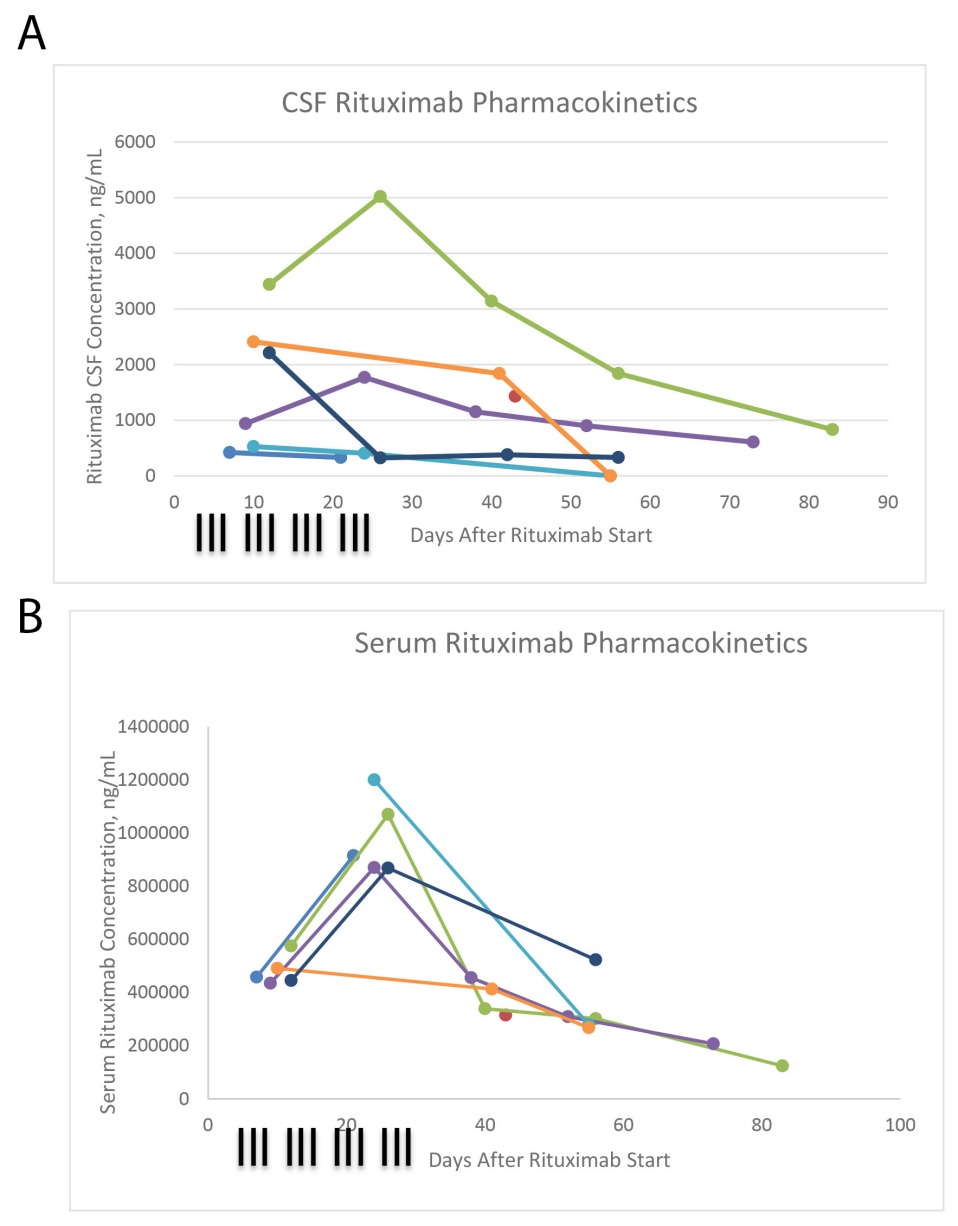
CSF rituximab concentrations **(A)**, serum rituximab concentrations **(B)**.

Serum and CSF rituximab concentrations are displayed in Figures 2A and 2B. Serum levels were comparable to those attained in thrice-weekly rituximab for CLL, administered on a similar schedule [19]. Rituximab was detected in the CSF of all patients in whom it was assayed. Rituximab persisted in both compartments past the cessation of systemic rituximab delivery, and through the sample of week 10 in 2 of 4 subjects who had complete data. Most CSF concentrations fell between 1% and 0.1% of serum levels [data not shown].

### Treatment at progression

In a post-hoc, exploratory analysis, data regarding the first subsequent therapy were obtained for 11 out of the 15 subjects who experienced progressive disease. At the time of first progression 4/11 subjects (36%) received radiation therapy. Six out of 11 (55%) received chemotherapy, and one (9%) received best supportive care alone.

## DISCUSSION

E1F05 demonstrates the activity of combined chemoimmunotherapy in patients with PCNSL in a multicenter study. There is growing evidence that the addition of the anti-CD20 antibody rituximab to high-dose methotrexate therapy is beneficial in patients with PCNSL.

Both PFS and OS in this study compare favorably with the results of RTOG 9310, upon which the cytotoxic chemotherapy backbone was based, while omitting radiation therapy. Median PFS is 34.1 months in E1F05, compared to 24 months in RTOG 9310, and median OS has not been reached versus 37 months in RTOG 9310 [22].

Many groups studying PCNSL have included rituximab as part of their induction regimen. Most informatively, a recently published interim analysis of the IELSG-32 randomized phase 2 trial confirmed that the addition of rituximab increased the ORR from 53% to 74%, and increased the rate of 2 year PFS from 36% to 46% [16]. Despite this growing consensus, there have been wide differences in the dosing schedule of rituximab, with most consisting of weekly or every-other week dosing [13-15, 23-25]. This study regimen of high dose rituximab was well tolerated: adverse events were mainly hematologic, and none precluded continuing therapy. There was one case of bowel perforation during the sixth week of the protocol in a subject who was also receiving steroids. The high early dosing resulted in measurable levels of rituximab in the CSF. CSF concentrations were highest at a time when serum concentrations were high, and when tumor volume was also high, creating a large surface area of incompetent blood brain barrier across which the large antibody can pass. While correlations between higher serum rituximab concentrations and response are reported in systemic B cell lymphomas [19], the optimal concentration or dose schedule in PCNSL is not known.

The study is limited by the single arm design and slow accrual. Radiation therapy was not part of the treatment regimen. Overt neurocognitive toxicity was not seen during the long follow up period, but formal neuropsychological testing was not performed. Long term comparative data answering this question are lacking. A randomized phase II study using a similar chemotherapy regimen in combination, with low dose WBRT given to responders in one of the arms, is currently underway; data from this study could help define the role of WBRT and the risk of neurotoxicity. The pre-specified accrual goal that would allow an informal comparison of complete response rates to a historical control was not met. However, the data are sufficiently mature to allow assessment of PFS. PFS for this chemoimmunotherapy regimen compares favorably to the same chemotherapy regimen given as combined modality treatment. These data support the inclusion of rituximab in PCNSL treatment regimens, and the deferral of radiation therapy as part of initial management.

## MATERIALS AND METHODS

### Subjects

Patients aged 18 or older with histologically documented non-Hodgkin lymphoma (NHL) confined to the central nervous system were eligible. Patients with an inconclusive biopsy, or patients who were not candidates for biopsy, were eligible provided that they had a typical CT or MRI scan of the brain (typical radiographic criteria are defined as the presence of a typical parenchymal contrast enhancing mass lesion(s)) and met at least one of two additional criteria: 1) A positive CSF cytology for lymphoma or a monoclonal lymphoid population as defined by cell surface markers or immunoglobulin gene rearrangement studies, or 2) Biopsy-proven involvement of the vitreous or uvea with cells apparent in the posterior chamber or vitreous on ophthalmological slit lamp examination. Patients all had bi-dimensionally measurable disease defined as a contrast enhancing tumor of at least 1 cm^2^ on the pretreatment MRI or CT scan. Subjects had not received any prior chemotherapy or radiation therapy for PCNSL; pre-enrollment steroid administration was allowed. Required features included an Eastern Cooperative Oncology Group performance status ≤ 3, and adequate bone marrow (absolute neutrophil count ≥ 1,500 /mm^3^, platelets ≥ 100,000 /mm^3^) liver (bilirubin ≤ institutional upper limit of normal (ULN), SGOT ≤ 2.0 x ULN) and kidney (creatinine clearance > 50 cc/min) function. Patients with a positive HIV serology, positive hepatitis B surface antigen, with pre-existing immunodeficiency, with evidence of systemic lymphoma, and/or if pregnant or nursing were excluded.

This study was approved by the institutional review boards of each participating institution and written informed consent was obtained from patients prior to study registration.

### On-study procedures

Physical exam was performed and performance status was assessed at baseline, every 2 weeks during treatment, 3 and 8 weeks post treatment, then every 3 months until 2 years from study registration, then every 6 months for subjects 2-5 years from study registration. Ophthalmologic exam, including slit lamp examination, was performed at baseline and 3 weeks after the completion of protocol treatment; if initial or subsequent exams were positive, an exam was performed additionally at weeks 5, 10, 14, and 8 weeks post treatment. Hematologic evaluations and liver function tests (LFTs) were assessed at baseline, every 2 weeks during treatment, and 3 weeks post treatment. Adverse events were assessed every 2 weeks during treatment using CTCAE version 4.0. Cranial MRI/CT was performed and neurologic function status was assessed at baseline, weeks 5,10, 14 during treatment, 3 and 8 weeks post treatment, then every 3 months until the subject was 2 years from study registration, then every 6 months if the subject was 2-5 years from study registration. All patients, including those who discontinued protocol treatment early, were to be followed for response until first progression and for survival for 5 years from study registration.

### Protocol treatment

Subjects received HD-MTX 3.5g/m2 with leucovorin rescue and vincristine 1.4mg/m2 IV (cap of 2.8mg) in weeks 1, 3, 5, 7, and 9; procarbazine 100mg/m2 PO daily for 7 days in weeks 1, 5, and 9; cytarabine 3g/m2 per day IV over 2 hours for 2 days on weeks 11 and 14; dexamethasone 16mg per day week 1, tapered by 4mg/week for weeks 2 and 3, then by 2 mg per week weeks 4-6; and rituximab 375mg/m2 IV infusion 3 times per week during weeks 1-4. Subjects with evidence of CSF involvement received intrathecal MTX 12mg every two weeks on weeks 2, 4, 6, 8, and 10 with leucovorin rescue.

### Statistical design and methods

The primary endpoint was complete response rate (CR). A two-stage design was used to minimize the number of patients treated with this regimen, which at the time of study design was minimal. If >23 out of a total of 39 eligible subjects achieved a CR (assuming >11 out of the first 23 eligible achieved a CR), the regimen would have been considered for further study. The probability of declaring the regimen promising was 90% if the true CR rate was 70% and 10% if the true CR rate was 50%. Allowing for a 10% ineligibility rate, the planned total accrual goal was 43 patients. Exact binomial confidences intervals (CI) were calculated for response.

Shortly after opening this study, new consensus criteria for determination of response in PCNSL were released by the International Primary CNS Lymphoma Collaborative Group [20]. These criteria distinguished complete response from an unconfirmed complete response (CRu; see “Abbreviations” below). For the purposes of the statistical plan, CR and CRu defined by the Collaborative Group were combined into “CR” previously defined by RECIST criteria.

Secondary endpoints included progression-free survival (PFS) and overall survival (OS). PFS was defined as time from study registration to first disease progression or death whichever occurred first, otherwise subject data were censored at time last known disease free. OS was defined as time from study registration to death, and otherwise censored at time last known alive. The Kaplan–Meier method was used to estimate PFS and OS distributions CIs for median outcome estimates were calculated via the method of Brookmeyer and Crowley. In situations where the upper limit of the CI was not reached, the last known censored time and a “+” sign was used. All eligible patients who began protocol treatment were included in baseline and outcome summaries, all patients who began protocol treatment were included in adverse event summaries.

### Documentation of response

Best overall response was graded according to the International PCNSL Collaborative Group response criteria [20]. In this system, CRu is complete response, unconfirmed, which means that either 1) a scan meets all requirements for a complete response but the subject continues to receive steroids; 2) a small enhancing abnormality remains where scar cannot be distinguished from disease; or 3) a persistent abnormality remains on ophthalmologic exam that is not consistent with lymphoma. Responses were graded according to a single central reviewer who was blinded to clinical data. Clinical decisions were based on incorporation of clinical data and local review of images.

### Pharmacokinetics

In subjects who received intrathecal therapy and who consented to pharmacokinetic studies, paired serum and CSF samples were collected. Samples were collected immediately prior to the 6^th^ infusion (week 2), immediately following the administration of the 12^th^ infusion (week 4), and during week 6, 8, and 10. The samples were assayed for rituximab concentrations at Genentech laboratories. The data were analyzed by the academic authors. Rituximab samples were processed and concentrations were determined according to procedures previously published [21].
